# MiR-125a-5p promotes osteoclastogenesis by targeting TNFRSF1B

**DOI:** 10.1186/s11658-019-0146-0

**Published:** 2019-03-28

**Authors:** Liang Sun, Jun Xiang Lian, Shu Meng

**Affiliations:** State Key Laboratory of Oral Diseases, National Clinical Research Center for Oral Diseases, West China Hospital of Stomatology, Sichuan University, No. 14, Section 3 of RenMinNanlu, Chengdu, 610041 Sichuan China

**Keywords:** miR-125a-5p, Osteoclast, Osteoporosis, TNFRSF1B

## Abstract

**Aim:**

To investigate the dysregulation of microRNAs (miRNAs) during the differentiation of osteoclasts and the precise roles of miR-125a-5p in the differentiation of osteoclasts.

**Methods:**

The cell model of RAW 264.7 osteoclast precursor cell differentiation induced by RANKL plus M-CSF stimulation was established. During the early stage of osteoclast differentiation, miRNA expression profiles were detected using the biochip technique and analyzed by cluster analysis. TargetScan, miRTarBase and miRDB database analysis was applied to find the key target genes of miR-125a-5p. A dual luciferase experiment was conducted to identify the direct target of miR-125a-5p. MiR-125a-5p mimic transfection and anti-miR-125-5p treatment were conducted to verify the role of miR-125q-5p in osteoclast differentiation. The levels of triiodothyronine receptor auxiliary protein (TRAP), matrix metallopeptidase 2 (MMP-2), MMP-9 and cathepsin K were analyzed by qRT-PCR and western blot assay. The expression levels of MMP-2 and MMP-9 were determined using western blotting and immunofluorescence assay. The migration and invasion of RAW 264.7 cells were assessed by wound healing and Transwell invasion assays. The proliferation of RAW 264.7 osteoclast precursor cells was detected using MTT assay.

**Results:**

There were 44 microRNAs differently expressed during the differentiation of RAW 264.7 osteoclast precursor cells into osteoclasts, 35 of which were up-regulated and 9 were down-regulated. By luciferase reporter assay, it was confirmed that the TNF receptor superfamily member 1B gene (TNFRSF1B) was the target gene of miR-125a-5p. Up-regulation of miR-125a-5p inhibited TNFRSF1B protein expression and promoted osteoclast differentiation whereas down-regulation of miR-125a-5p caused completely opposite results.

**Conclusions:**

In conclusion, overexpression of miR-125a-5p suppresses the expression of TNFRSF1B and promotes osteoclast differentiation. These results reveal the crucial role of miR-125a-5p in the differentiation of osteoclasts.

## Background

Osteoclasts are derived from monocyte-macrophage precursors that arise from multipotent hematopoietic stem cells [1]. Many cytokines and exogenous hormones have been identified to be involved in osteoclastogenesis through transcription factors that positively or negatively modulate osteoclast proliferation, survival, differentiation and function. Colony stimulating factor 1 (M-CSF) and receptor activator of nuclear factor kappa-B ligand (RANKL), both produced by osteoblast and activated T cells, are significant cytokines for osteoclastogenesis [2]. RANKL induces the expression of nuclear factor of activated T-cells cytoplasmic (NFATc1). Osteoclast-specific markers, such as tartrate-resistant acid phosphatase (TRAP) and cathepsin K, have multiple sites recognized by NFATc1, a vital transcription factor during osteoclastogenesis.

MicroRNAs (miRNAs), which are small non-coding RNAs of ~ 22 nucleotides, play important roles in post-transcriptional regulation in various cellular processes [3–5]. MiRNAs contribute to every process of osteogenesis from embryonic bone development to maintenance of tissues, through regulating the growth, differentiation and functional activities of osteoblasts, osteocytes and osteoclasts. Various miRNAs have been reported to regulate osteoblastic differentiation, proliferation and bone formation [6, 7]. Recently, the function of miRNAs in osteoclastic differentiation and activity has begun to be elucidated. MiRNA-125a-5p (miR-125a-5p), a type of newly discovered miRNA molecule, has been verified to be involved in the progression of various cancers, including hepatocellular carcinoma, laryngeal cancer, lung cancer and prostate cancer [8–11]. The tumor suppressive function of miR-125a-5p has also been demonstrated by a number of studies on a variety of tumor types. Notably, miR-125a has been shown to enhance the sensitivity of paclitaxel-resistant colon cancer cells to paclitaxel, which supports the application miR-125a as a novel ancillary drug for use in chemotherapy for patients with tumors [12]. Recent studies have suggested that miR-125a-3p regulates the expression of G protein-coupled receptor kinase interacting protein 1 (GIT1) and inhibits osteoblastic proliferation and differentiation [13]. However, the expression and function of miR-125a-5p in osteoclasts remain elusive.

In this study, we found that miR-125a-5p was overregulated in the process of M-CSF and RANKL induced osteoclastogenesis. Up-regulation of miR-125a-5p increased the osteoclast activity of RAW 264.7 osteoclast precursor cells and down-regulation of miR-125a-5p significantly reduced osteoclast activity. In addition, we demonstrated that miR-26a acted as a positive regulator of osteoclast formation by inhibiting the expression of TNFRSF1B.

## Methods

### Cell culture

The RAW 264.7 and 293T cells were obtained from the Chinese Academy of Sciences Cell Bank of Type Culture Collection (CBTCCCAS, Shanghai, China). RAW 264.7 and 293T cells were maintained in Dulbecco’s modified Eagle’s medium (DMEM, Hyclone, Logan, Utah, USA) containing 10% FBS, 100 μg/mL streptomycin and 100 μg/mL penicillin. RAW 264.7 cells were induced with M-CSF (50 ng/ml) and RANKL (50 ng/ml) for osteoclastogenesis.

### MiRNA microarray assay

Total RNA was extracted from RAW 264.7 cells that were treated with or without RANKL for 3 days. MiRNAs were isolated and labeled using either Cy3 or Cy5. MiRNA microarray assay was completed by Phalanx Biotech Group (Xinzhu, Taiwan, China). The probe content was in accordance with Version 19.0 of the Sanger miRBase database. The signal intensity of each spot was calculated by the program R (2.12.1). The median value of repeating spots was selected for future analysis. We filtered out the spots which flagged < 0 within the array. Spots that passed the criteria were normalized by the invariant set normalization method. Normalized spot intensities were transformed to gene expression log2 ratios between the control group and treatment group using the pair-wise *t* test. The spots with a |log2 ratio| ≥ 0.585 and a *p* value < 0.05 were selected for analysis.

### TRAP staining assay

Briefly, after 3 days of culture, RANKL and M-CSF-induced RAW 264.7 cells were fixed by immersing in fixative solution for 30 s at room temperature and then rinsed in deionized water. Then, TRAP staining fluid was added, and the plate was incubated at 37 °C protected from light for 1 h. After removal of the TRAP solution, the plate was washed three times using distilled water. The TRAP positive staining multinuclear cells were recorded using a Zeiss inverted microscope (Carl Zeiss, Hallbergmoos, Germany).

### Transient transfection

MiR-125a-5p mimics and anti-miR-125a-5p were synthesized by Sangon Biotech (Shanghai, China). The TNFRSF1B expression construct was generated by subcloning PCR-amplified full-length human TNFRSF1B cDNA into the pcDNA3.1(+) plasmid. The siRNA pool against TNFRSF1B was synthesized from Shanghai GenePharma, Co., Ltd. (Shanghai, China). Transfection was conducted using Lipo2000 Transfection Reagent (Beyotime, Nanjing, Jiangsu, China) according to the instructions. After 24 h, the transfected cells were collected for experimental determination.

### MiRNA extraction

Total RNA was extracted using the miRNeasy Mini Kit (QIAGEN, Hilden, Germany). Cells were lysed using 700 μl of QIAzol and mixed with 140 μl of chloroform. After being centrifuged at 12,000 g for 15 min at 4 °C, the upper aqueous phase was transferred into another RNeasy Mini spin column in a 2 ml collection tube and mixed with 100% ethanol. After being washed using 700 μl of Buffer RWT and 500 μl Buffer RPE, RNA was collected for future real-time PCR assay.

### MTT assay

The cell viability was assessed using the 3-(4,5-dimethylthiazol-2-yl)-2,5-diphenyltetrazolium bromide (MTT) assay. 4 × 10^3^ cells were cultured into 96-well plates and cultured for the indicated time. 10 μL of MTT (5 mg/mL; Sigma-Aldrich, St. Louis, MI, USA) was added into 96-well plates and then incubated for another 4 h at 37 °C. Then, 200 μl of dimethyl sulfoxide (DMSO) was added to the 96-well plates. Finally, the absorbance was measured at 450 nm using a Synergy HT Multi-Mode Microplate Reader (Bio-Tek, Winooski, VT, USA).

### Wound healing assay

First, cells were cultured in six-well plates for 24 h. Wounds were scratched using a 20 μl pipette tip. Then, plates were washed with fresh medium to remove the non-adherent cells. Then, cells were cultured for 0 or 24 h, and then photographed [14].

### Transwell invasion assay

Transwell chambers (24-well Transwell chambers, 8-μm pore size; Corning, Inc., Corning, NY, USA) were used for the invasion assay. The Transwell membrane was precoated with 1:4 diluted Matrigel. A 200 μl cell suspension (10^5^/ml) was added to the upper chamber. The medium containing 10% FBS was added to the lower chamber. After 24 h, the invaded cells were fixed using 4% paraformaldehyde, stained with 0.1% crystal violet, and counted from five random fields by bright field microscopy [15].

### Quantitative real-time PCR (qRT-PCR)

The total RNA was extracted with TRIzol Reagent (Invitrogen, Carlsbad CA, USA). 0.5 μg of RNA was reverse transcribed using PrimeScript RT reagent Kit (TakaraBio, Tokyo, Japan) according to the manufacturer’s instructions. The Stem-loop RT-PCR was applied for the quantification of miR-125a-5p. Two microliters of cDNA were used for detecting the level of mRNA and miRNA using quantitative PCR using the SYBR Premix Ex TaqTMII Kit (TakaraBio, Tokyo, Japan). GAPDH and U6 were used as a normalization control for mRNA and miRNA. Primers used were synthesized by Beijing Sunbiotech Co., Ltd. (Beijing, China) and their sequences were as follows: GAPDH (forward: 5′-TGGATTTGGACGCATTGGTC-3′ and reverse: 5′-TTTGCACTGGTACGTGTTGAT-3′), TNFRSF1B (forward: 5′-CGGGCCAACATGCAAAAGTC-3′ and reverse: 5′-CAGATGCGGTTCTGTTCCC-3′), ACP5 (forward: 5′-GACTGTGCAGATCCTGGGTG-3′ and reverse: 5′-GGTCAGAGAATACGTCCTCAAAG-3′), MMP-9 (forward: 5′-TGTACCGCTATGGTTACACTCG-3′ and reverse: 5′-GGCAGGGACAGTTGCTTCT-3′), MMP-2 (forward: 5′-TGACTTTCTTGGATCGGGTCG-3′ and reverse: 5′-AAGCACCACATCAGATGACTG-3′), TRAP (forward: 5′-TCACCCTGACCTATGGTGC-3′ and reverse: 5′-GCCGGACTCCAATGTTAAAGC-3′) cathepsin K (forward: 5′-CTGGCTGGGGTTATGTCTCAA-3′ and reverse: 5′-GGCTACGTCCTTACACACGAG-3′).

### Luciferase reporter assay

293T cells (1 × 10^6^) were seeded into 6-well plates. Then, cells were transfected with pGL3-TNFRSF1B 3′-UTR (Addgene) and pRL-TK (Promega, Madison, WI, USA) Renilla luciferase plasmid with miR-125a-5p, anti-miR-125a-5p or NC following the manufacturer’s instructions (Lipofectamine RNAiMAX, Invitrogen, USA). Luciferase assays were performed with the dual-luciferase reporter assay system (Promega, Madison, WI, USA) according to the manufacturer’s instructions. Luminescent signals were quantified by a luminometer (Glomax, Promega, Madison, WI, USA), and each value from the firefly luciferase construct was normalized by Renilla luciferase assay.

### Western blot analysis

Cells were lysed using the lysis buffer and protease inhibitor cocktail on ice for 30 min. Total protein were collected by centrifugation at 15,000 g at 4 °C for 30 min and then subjected to SDS-PAGE and transferred to polyvinylidene difluoride (PVDF) membrane. The membrane was blocked with 5% BSA and incubated with antibodies against TRAP (Santa Cruz Biotechnology, Dallas, Texas, USA), TNFRSF1B (Santa Cruz Biotechnology, Dallas, Texas, USA), MMP-2 (Cell Signaling Technology, Danvers, MA, USA), MMP-9 (Cell Signaling Technology, Danvers, MA, USA), ACP5 (Cell Signaling Technology, Danvers, MA, USA), cathepsin K (Cell Signaling Technology, Danvers, MA, USA), and GAPDH (Santa Cruz Biotechnology) followed by incubation with horseradish peroxidase-conjugated IgGs (1∶10,000, Bioworld Biotechnology, Nanjing, Jiangsu, China). The target proteins were assessed using an ECL system (Millipore, Braunschweig, Germany) and were visualized by the ChemiDoc XRS system (Bio-Rad, Hercules, CA, USA) [16].

### Immunofluorescence

RAW 264.7 cells were fixed using pre-cold acetone, and then rinsed with PBS three times. The cells were permeabilized in 0.1% Triton X-100 and incubated with 1% BSA/PBS. Then, cells were immunostained by incubating with antibody against MMP-2, MMP-9 or TNFRSF1B (diluted 1:500, Santa Cruz Biotechnology, Dallas, Texas, USA) overnight at 4 °C. After being washed with PBS, cells were incubated with the FITC-conjugated goat anti-rabbit secondary antibody (diluted 1:60, Boster Biotechnology, Wuhan, Hubei, China). Nuclei were stained with DAPI (Biotime Biotech, Haimen, Jiangsu, China). Images were taken on a Zeiss inverted microscope (Carl Zeiss, Hallbergmoos, Germany).

### Statistical analysis

Data are presented as mean ± SD per experimental condition unless noted otherwise. The data were presented as mean ± SD. Differences in the results of two groups were evaluated using either two-tailed Student’s *t* test or one-way ANOVA followed by post hoc Dunnett’s test. The differences with *P* < 0.05 were considered statistically significant.

## Results

### MiR-125a-5p is up-regulated in osteoclastogenesis induced by M-CSF and RANKL

**Fig. 1:**
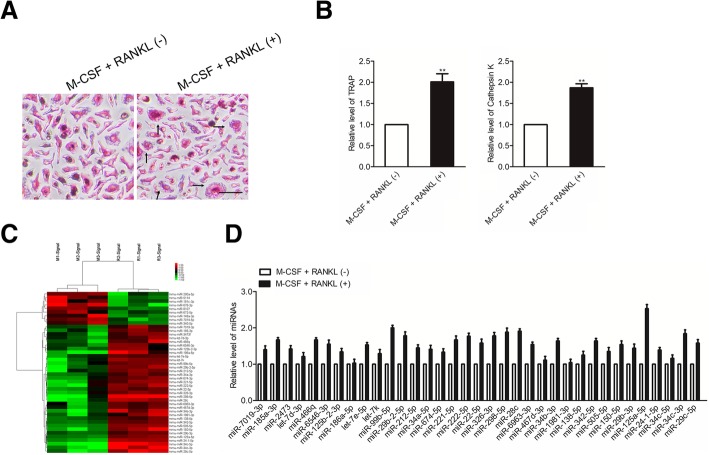
MiR-125a-5p was upregulated during osteoclastogenesis. **a** RAW 264.7 cells were cultured in the absence or the presence of M-CSF (50 ng/ml) and RANKL (50 ng/ml) for 72 h. TRAP-positive (pink to purple) multinucleated cells with more than three nuclei were counted as mature osteoclasts; **b** RAW 264.7 cells were cultured in the absence or the presence of M-CSF (50 ng/ml) and RANKL (50 ng/ml) for 0 h or 72 h respectively. The relative expression of TRAP and cathepsin K was detected by qRT-PCR. ^**^ *P* < 0.01 versus the group without stimulation of M-CSF and RANKL; **c** Microarray assays were performed in RAW 264.7 cells with or without M-CSF and RANKL induction for 3 days. Red and green indicate high expression levels and low expression levels, respectively; **d** Relative miRNA levels in M-CSF and RANKL-induced RAW 264.7 cells were analyzed by qRT-PCR, miRNA levels were normalized to U6. The data represent the mean ± SD of 3 experiments in triplicate. ^**^ *P* < 0.01 compared to the group without stimulation of M-CSF and RANKL

M-CSF and receptor activator of RANKL are two critical cytokines involved in osteoclast differentiation. To investigate the effect of RANKL and M-CSF on osteoclast differentiation, RAW 264.7 osteoclast precursor cells were cultured in the absence or the presence of M-CSF (50 ng/ml) and RANKL (50 ng/ml) for 72 h. TRAP staining analysis revealed that RAW 264.7 cells stimulated with M-CSF and RANKL were differentiated into more TRAP-positive osteoclasts than those without M-CSF and RANKL treatment (Fig. 1a). Consistently, the levels of TRAP and cathepsin K were significantly higher in the RAW 264.7 cells stimulated with M-CSF and RANKL than the control cells (Fig. 1b). All these results indicate that initiating treatment with M-CSF and RANKL may serve as a condition for osteoclastogenesis. To investigate the miRNAs involved in osteoclastogenesis, microarray assays were performed. RAW 264.7 cells were induced by treatment with M-CSF and RANKL. RAW 264.7 cells were cultured without M-CSF and RANKL as controls. Our results showed that 44 miRNAs were differently expressed during the differentiation of RAW 264.7 cells into osteoclasts (Fig. 1c). Thirty-five miRNAs were significantly increased. Conversely, nine miRNAs were down-regulated. The expression of 35 upregulated miRNAs was further confirmed by qRT-PCR; the results were consistent with those of miRNA microarray. Among them, miR-125a-5p was the most obviously up-regulated miRNA after RAW 264.7 cells were stimulated by M-CSF and RANKL (Fig. 1d). The results suggest that miR-125a-5p plays an important role in osteoclastogenesis.

### MiR-125a-5p is essential to osteoclastogenesis

**Fig. 2:**
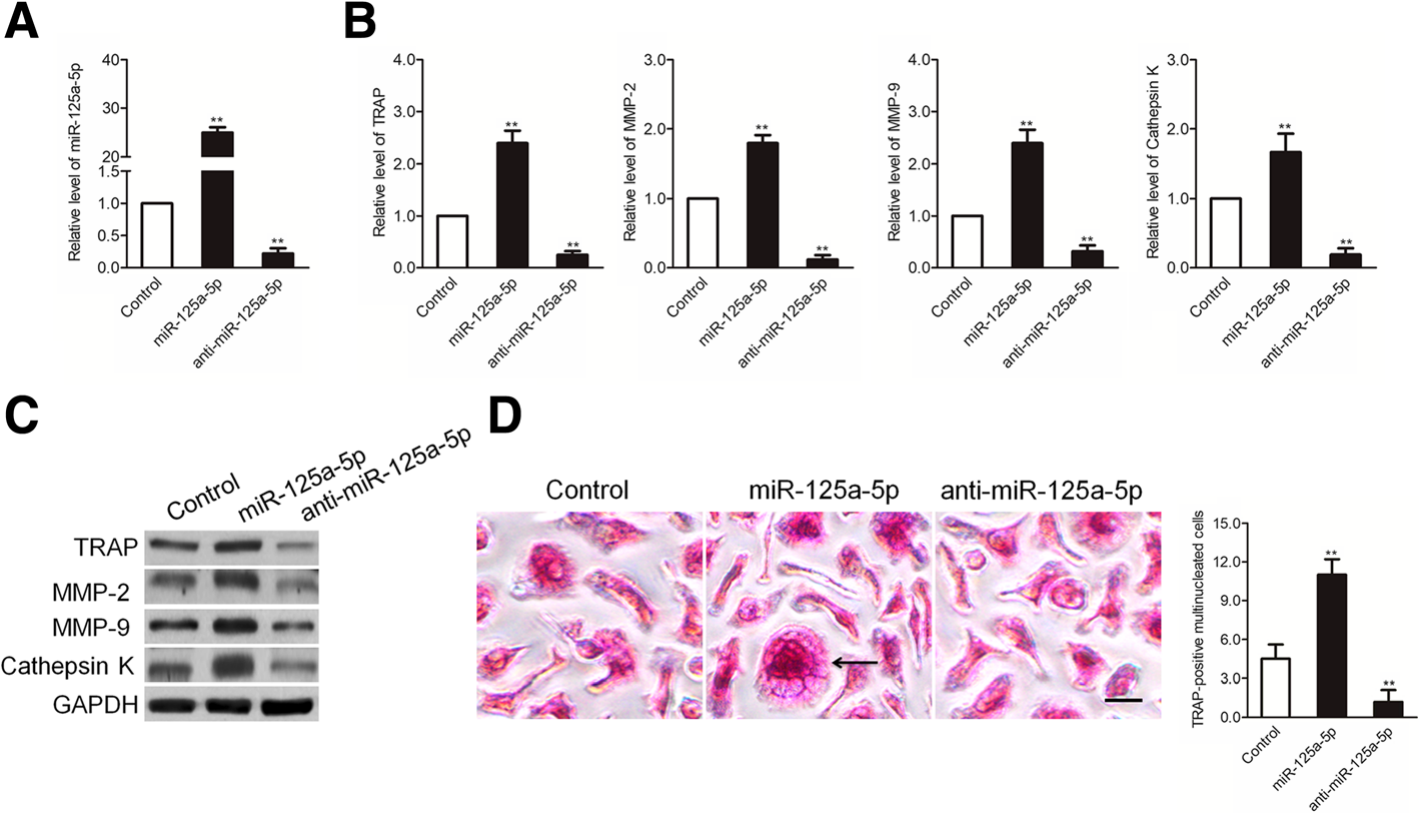
MiR-125a-5p regulates osteoclastogenesis from RAW 264.7 cells. **a** RAW 264.7 cells were induced with M-CSF and RANKL for 3 days. The relative miR-125a-5p levels were determined by qRT-PCR after treatment with miR-125a-5p mimics or anti-miR-125a-5p; **b** Relative TRAP, MMP-2, MMP-9, and cathepsin K mRNA levels were determined by qRT-PCR and normalized to GAPDH; **c** The expression levels of TRAP, MMP-2, MMP-9, and cathepsin K were determined by western blotting assay; **d** Representative photographs of osteoclastogenesis and the number of TRAP-positive multinucleated cells are shown. The data represent the mean ± SD of three experiments in triplicate. ^**^ *P* < 0.01 compared with control

To assess the significance of miR-125a-5p for osteoclast differentiation, we treated RAW 264.7 cells with miR-125a-5p mimics or anti-miR-125a-5p in the course of osteoclastogenesis. The results showed that miR-125a-5p levels are substantially upregulated by miR-125a-5p mimic treatment and markedly downregulated by anti-miR-125a-5p treatment (Fig. 2a). TRAP, MMP-2, MMP-9 and cathepsin K are important markers for the differentiation and activity of osteoclasts. After 3 days of induction, the expression of TRAP, MMP-2, MMP-9 and cathepsin K was significantly up-regulated by miR-125a-5p mimics and down-regulated by anti-miR-125a-5p compared to controls (Fig. 2b). Consistently, the western blotting assay demonstrated that up-regulation of miR-125a-5p mimics increased the expression of TRAP, MMP-2, MMP-9 and cathepsin K and down-regulation of miR-125a-5p caused opposite results (Fig. 2c). Functionally, the number of TRAP-positive, multinucleated osteoclasts per well was substantially increased in the miR-125a-5p mimic treatment group and markedly reduced in the anti-miR-125a-5p treatment group compared to the negative control group (Fig. 2d). These results demonstrate that miR-125a-5p is essential for osteoclastogenesis.

### MiR-125a-5p promotes the proliferation and motility of osteoclast precursors

**Fig. 3:**
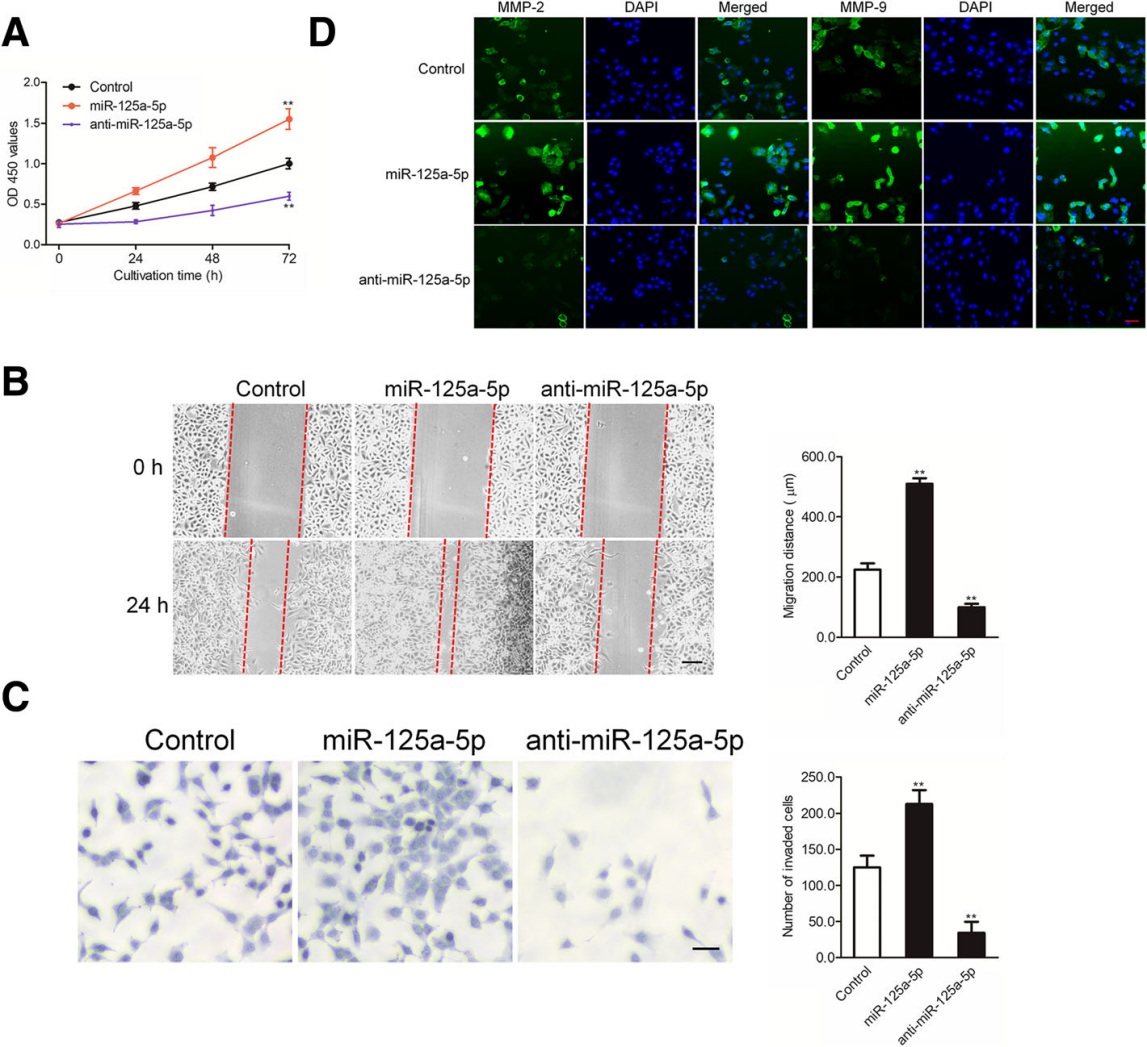
MiR-125a-5p increases osteoclast precursor proliferation, motility and invasion. **a** Cells were cultured for 3 days in the presence of M-CSF and RANKL. The proliferation of RAW 264.7 cells was determined by MTT assay; **b** The migration of osteoclast precursors was measured using wound healing assay. Scale bars indicate 100 μm; **c** The invasion of RAW 264.7 cell was determined using Transwell invasion assay. Data are mean ± SD, representative of at least three independent experiments performed in triplicate; **d** The expression of MMP-2 and MMP-9 of RAW 264.7 cells treated with miR-125a-5p or anti-miR-125a-5p was analyzed by immunofluorescence assay. ** *P* < 0.01 versus control

The proliferation and motility of osteoclast precursors are crucial for these cells to develop into fully mature osteoclasts and function properly. To further assess whether miR-125a-5p plays a role in these processes, RAW 264.7 cells were treated with miR-125a-5p or anti-miR-125a-5p and then stimulated with M-CSF and RANKL. MiR-125a-5p treatment significantly increased the proliferation of osteoclast precursors compared with control cells (Fig. 3a). The migration and invasion of RAW 264.7 cells were estimated after treatment with miR-125a-5p mimics or anti-miR-125a-5p followed by incubation with M-CSF and RANKL. The migration and invasion of RAW 264.7 were increased in miR-125a-5p-treated cells (Fig. 3b-c). Furthermore, the expression of MMP-2 and MMP-9 was consistently higher in miR-125a-5p-treated cells and lower in cells that were treated with anti-miR-125a-5p (Fig. 3d).

### MiR-125a-5p directly targets TNFRSF1B

**Fig. 4:**
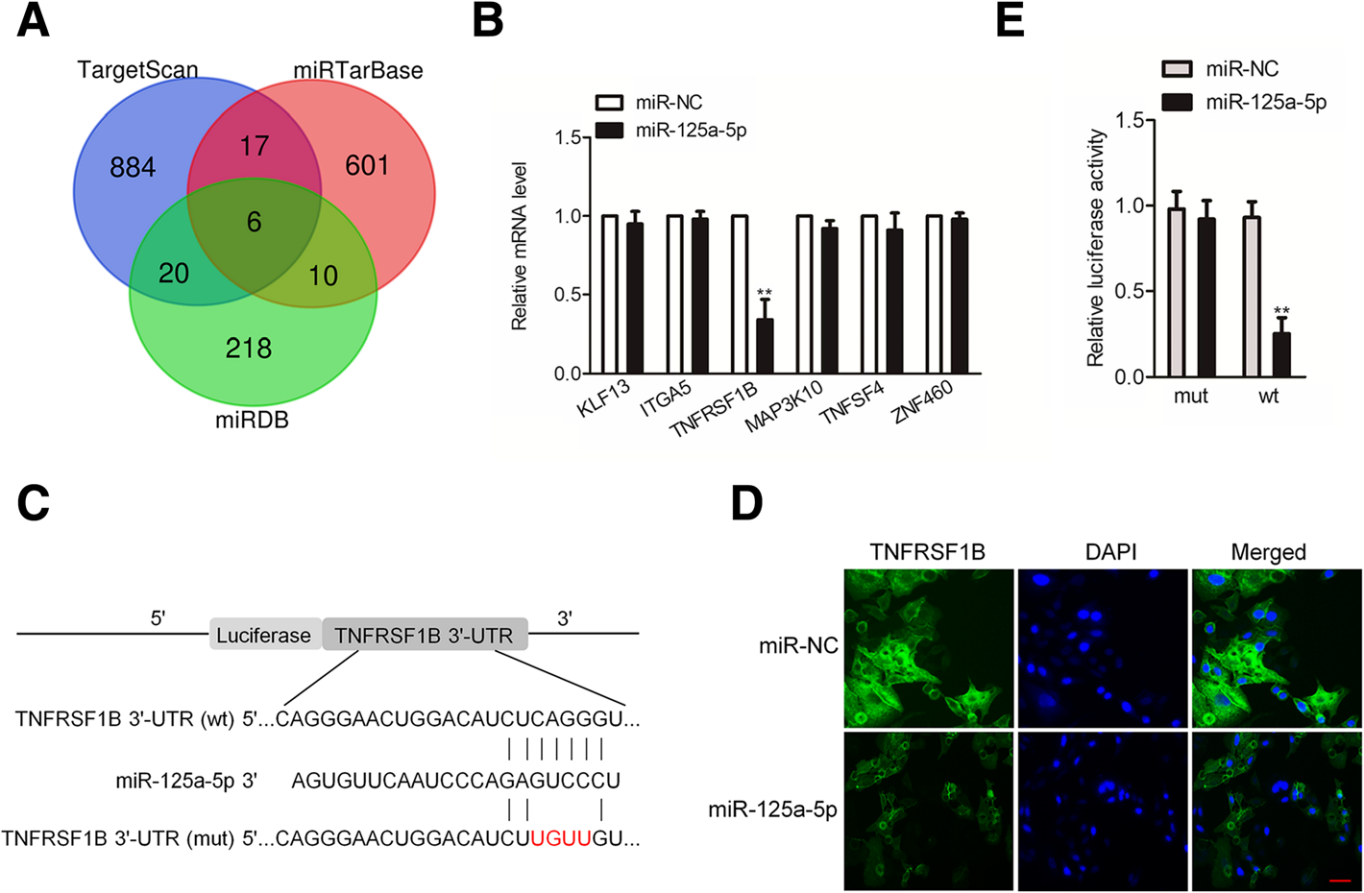
MiR-125a-5p directly regulates the expression of TNFRSF1B in RAW 264.7 cells. **a** The complementary sequences of miR-125a-5p were discovered in 3′-UTR of TNFRSF1B mRNA using TargetScan, miRTarBase and miRDB. Venn graph represents the number of candidate common target genes determined by three bioinformatics analyses. **b** RAW 264.7 cells were transfected with miR-NC or miR-125a-5p and the levels of potential target genes were measured by qRT-PCR assay. **c** The complementary sequences of miR-125a-5p were discovered in 3′-UTR of TNFRSF1B mRNA using TargetScan. The mutagenesis was performed in the complementary sites for the seed region of miR-125a-5p (wt, wild type; mut, mutant type). **d** RAW 264.7 cells were transfected with miR-125a-5p or miR-NC. Immunofluorescence assay indicated that up-regulation of miR-125a-5p reduced the expression of TNFRSF1B. **e** MiR-125a-5p inversely modulated the luciferase activity of plasmids containing wt 3′-UTR of TNFRSF1B. ^**^ *P* < 0.01 compared to miR-NC

Then, the targets of miR-125a-5p were predicted using online analysis tools, including TargetScan (http://www.targetscan.org), miRTarBase (http://mirtarbase.mbc.nctu.edu.tw/php/index.php) and miRDB (http://www.mirdb.org/) [17]. The six common target genes that were obtained from three bioinformatics analysis tools are summarized in Fig. 4a. In order to identify the direct gene of miR-125a-5p, the mRNA levels of these genes in miR-125a-5p or miR-NC transfected RAW 264.7 cells were detected using qRT-PCR assay. As shown in Fig. 4b, the mRNA level of TNFRSF1B was significantly inhibited by miR-125a-5p in RAW 264.7 cells. The 3′-UTR of TNFRSF1B containing the complementary binding sites within miR-125a-5p is shown in Fig. 4c. In addition, up-regulation of miR-125a-5p reduced the protein expression of TNFRSF1B in RAW 264.7 cells (Fig. 4d). Notably, the luciferase activity of 293T cells that were transfected wild type (wt) 3′-UTR of TNFRSF1B was reduced by miR-125a-5p, while the luciferase activity of 293T cells that were transfected with mutated type (mut) 3′-UTR of TNFRSF1B was not affected by miR-125a-5p (Fig. 4e). All these findings suggest that miR-125a-5p is a negative regulator of TNFRSF1B.

### TNFRSF1B is needed for miR-125a-5p to regulate the growth, migration and invasion of RAW 264.7 cells

**Fig. 5:**
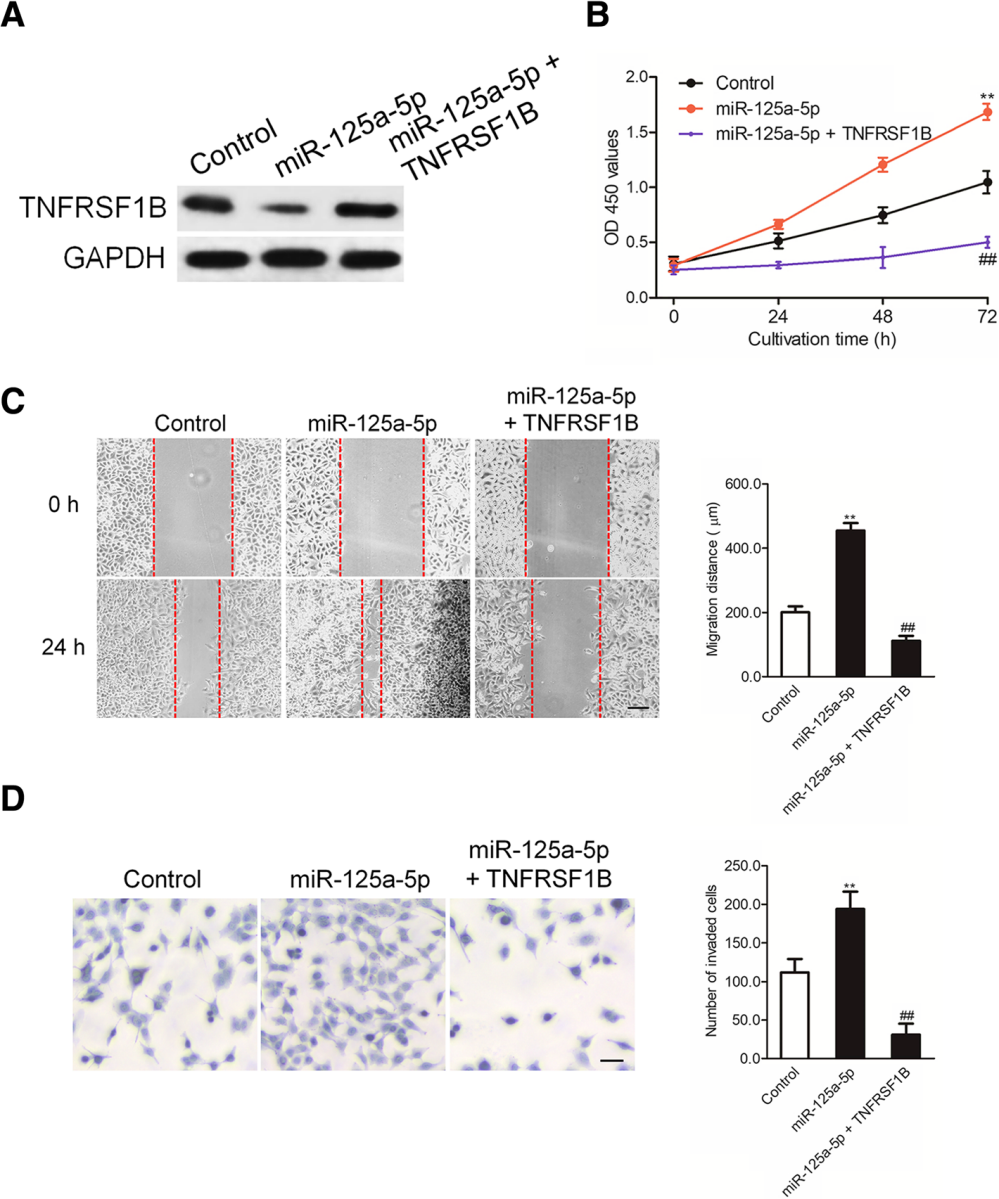
Over-expression of TNFRSF1B reverses the effect of miR-125a-5p in RAW 264.7 cells. **a** RAW 264.7 cells that were transfected with miR-125a-5p and TNFRSF1B were subjected to immunoblotting assay. **b** RAW 264.7 cells were transfected with miR-125a-5p alone or miR-125a-5p in combination with TNFRSF1B. The proliferation of RAW 264.7 cells was detected using MTT assay. **c** Migration of RAW 264.7 cells was determined by wound healing assay. **d** RAW 264.7 cells were transfected with miR-125a-5p alone or miR-125a-5p in combination with TNFRSF1B. The invasion of RAW 264.7 cells was measured by Transwell invasion assay. ^**^ *P* < 0.01 compared to control, ^##^ *P* < 0.01 compared to miR-125a-5p

Previous results demonstrated that TNFRSF1B is the target gene of miR-125a-5p in RAW 264.7 cells. Then, we performed rescue experiments to further demonstrate that TNFRSF1B was needed for miR-125a-5p to regulate the phenotypes of RAW 264.7 cell. RAW 264.7 cells were transfected with miR-125a-5p alone or cotransfected with miR-125a-5p and pcDNA3.1(+) containing TNFRSF1B. The expression of TNFRSF1B was analyzed by western blotting assay using anti-TNFRSF1B antibody (Fig. 5a). Then, the proliferation, migration and invasion of RAW 264.7 cells were investigated. As shown in Fig. 5b, over-expression of TNFRSF1B decreased the proliferation of RAW 264.7 cells that were promoted by miR-125a-5p transfection. Similarly, over-expression of TNFRSF1B significantly decreased the migration and invasion abilities of RAW 264.7 cells that were increased by miR-125a-5p (Fig. 5c-d). These findings indicate that miR-125a-5p promotes the growth, migration and invasion of RAW 264.7 cells through targeting TNFRSF1B.

### Confirmation that miR-125a-5p regulates the phenotypes of RAW 264.7 cells dependent on TNFRSF1B

**Fig. 6:**
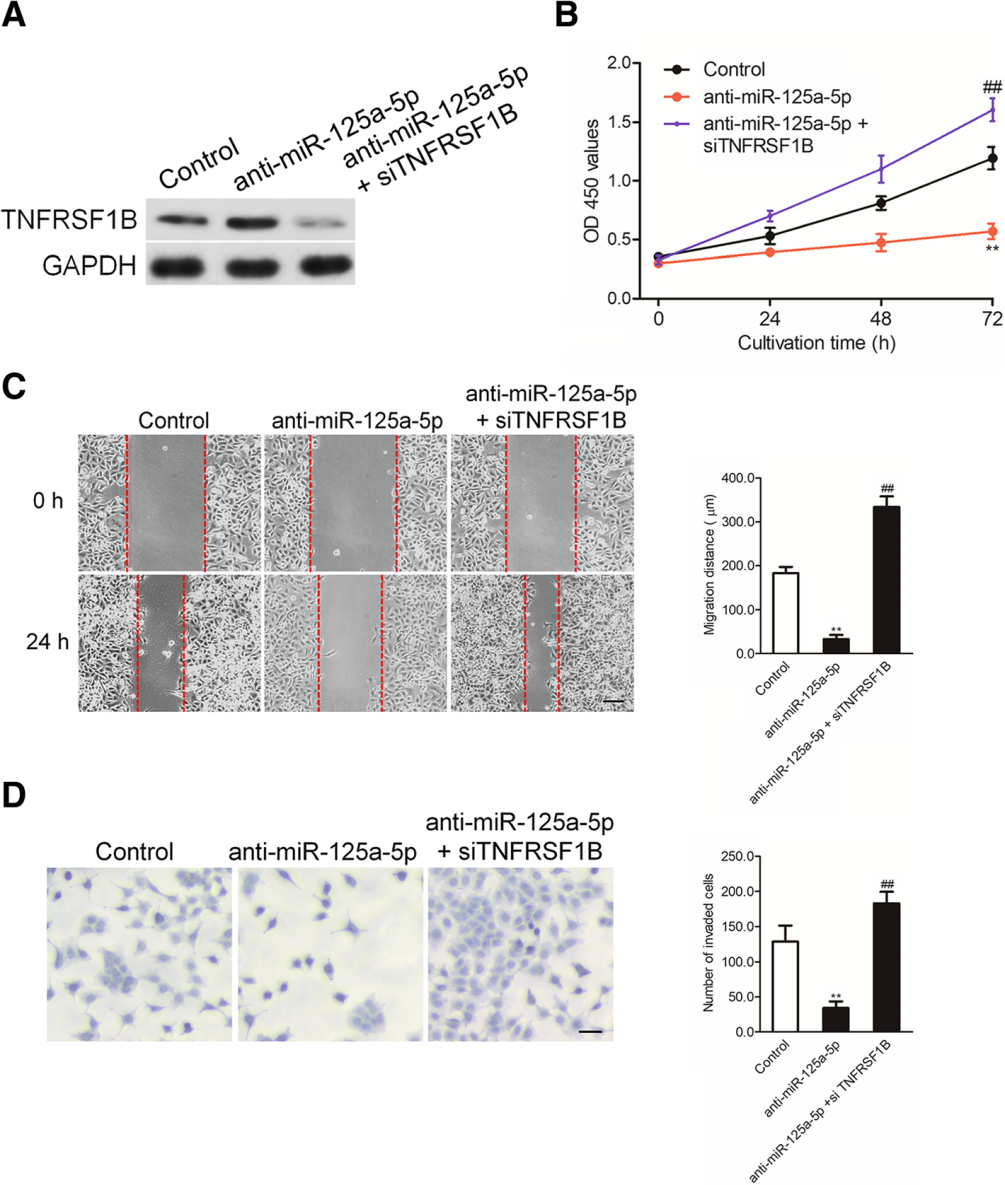
Down-expression of TNFRSF1B reverses the effect of miR-125a-5p in RAW 264.7 cells. **a** RAW 264.7 cells that were treated with anti-miR-125a-5p and siTNFRSF1B were subjected to immunoblotting assay. **b** RAW 264.7 cells were treated with anti-miR-125a-5p alone or anti-miR-125a-5p in combination with siTNFRSF1B. The proliferation of RAW 264.7 cells was detected using MTT assay. **c** The migration of RAW 264.7 cells was determined by wound healing assay. **d** RAW 264.7 cells were treated with anti-miR-125a-5p alone or anti-miR-125a-5p in combination with siTNFRSF1B. The invasion of RAW 264.7 cells was analyzed by Transwell invasion assay. ^**^ *P* < 0.01 compared to control, ^##^ *P* < 0.01 compared to anti-miR-125a-5p

Finally, we conducted another rescue experiment to prove that miR-125a-5p inhibition of the aggressive phenotypes of RAW 264.7 cells was dependent on regulating TNFRSF1B. RAW 264.7 cells were treated with anti-miR-125a-5p alone or co-treated with anti-miR-125a-5p and siRNA targeting TNFRSF1B. The protein expression of TNFRSF1B was assessed by immunoblotting assay (Fig. 6a). Then, the proliferation, migration and invasion abilities of RAW 264.7 cell were investigated. As shown in Fig. 6b, down-expression of TNFRSF1B obviously increased the proliferation of RAW 264.7 cells that was inhibited by anti-miR-125a-5p. Similarly, down-expression of TNFRSF1B dramatically promoted the migration and invasion abilities of RAW 264.7 cells that was suppressed by anti-miR-125a-5p (Fig. 6c-d). These results indicate that miR-125a-5p regulates the growth, migration and invasion of RAW 264.7 cells through targeting TNFRSF1B.

### MiR-125a-5p promotes osteoclast differentiation through targeting TNFRSF1B

**Fig. 7:**
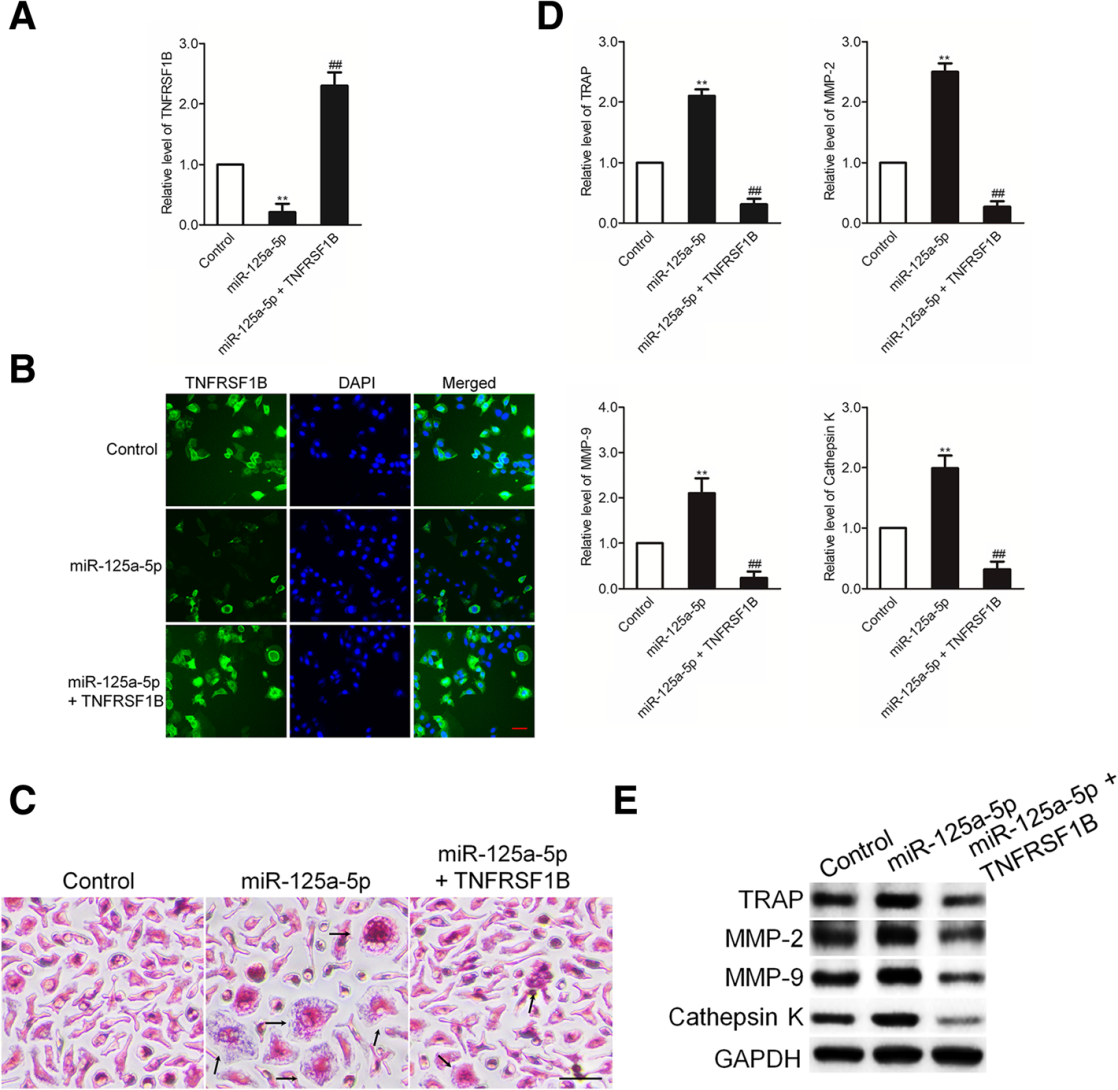
MiR-125a-5p promotes osteoclast differentiation through targeting TNFRSF1B. **a** RAW 264.7 cells were treated with miR-125a-5p mimics or miR-125a-5p mimics with pcDNA3.1(+) containing TNFRSF1B. The expression of TNFRSF1B was assessed using qRT-PCR assay; **b** RAW 264.7 cells were treated with miR-125a-5p mimics or miR-125a-5p mimics with pcDNA3.1(+) containing TNFRSF1B. The expression of TNFRSF1B was assessed using immunofluorescence assay; **c** RAW 264.7 cells were treated with miR-125a-5p mimics or miR-125a-5p mimics with pcDNA3.1(+) containing TNFRSF1B in the presence of M-CSF + RANKL. TRAP-positive (pink to purple) multinucleated cells with more than three nuclei were counted as mature osteoclasts; **d** RAW 264.7 cells were treated with miR-125a-5p mimics or miR-125a-5p mimics with pcDNA3.1(+) containing TNFRSF1B. The mRNA levels of TRAP, MMP-2, MMP-9, and cathepsin K were determined using qRT-PCR assay; **e** The protein expression of TRAP, MMP-2, MMP-9, and cathepsin K in RAW 264.7 cells was determined by western blot assay. ^**^ *P* < 0.01 compared to control, ^##^ *P* < 0.01 compared to miR-125a-5p

It has been demonstrated that TNFRSF1B regulates RANKL-induced osteoclast differentiation from RAW 264.7 osteoclast precursors. To test the effect of miR-125a-5p on TNFRSF1B during osteoclast differentiation, RAW 264.7 cells were treated with miR-125a-5p mimics or miR-125a-5p mimics with pcDNA3.1(+) containing TNFRSF1B. The expression of TNFRSF1B was determined by qRT-PCR and immunofluorescence assay. As shown in Fig. 7a-b, the level of TNFRSF1B was significantly inhibited by miR-125a-5p and increased by the over-expression of TNFRSF1B. Importantly, up-regulation of miR-125a-5p promoted the osteoclastogenesis of RAW 264.7 cells dependent on TNFRSF1B (Fig. 7c). Therefore, we further detected the levels of TRAP, MMP-2, MMP-9, and cathepsin K in RAW 264.7 cells using qRT-PCR and western blotting assays. The results demonstrated that the expression of TRAP, MMP-2, MMP-9, and cathepsin K was up-regulated in RAW 264.7 cells that were transfected with miR-125a-5p after M-CSF + RANKL induction and over-expression of TNFRSF1B neutralized the promoter effect of miR-125a-5p on the expressions of TRAP, MMP-2, MMP-9, and cathepsin K (Fig. 7d-e). All these data suggest that miR-125a-5p regulates M-CSF + RANKL-activated osteoclast differentiation of RAW 264.7 cells by targeting TNFRSF1B.

## Discussion

Accumulating evidence has revealed that osteoclast differentiation is regulated by many cytokines and transcription factors, such as colony stimulating factor 1 (M-CSF), receptor activator of nuclear factor kappa-B ligand (RANKL), Src, Myc, nuclear factor (NF)-κB, nuclear factor of activated T cells 1 (NFATc1), and TNFRSF1B [18]. M-CSF has been reported to be essential for osteoclast differentiation via binding to its receptor c-Fms [19]. RANKL plays a prominent role in regulating osteoclast formation from mononuclear precursors by interacting with its receptor RANK. Until present, osteoclast differentiation can be achieved with macrophages in the stimulation of M-CSF and RANKL [20].

A large number of miRNAs play critical roles in modulating various biological processes of osteoclast differentiation and function [21]. Generally, miRNAs that decrease in expression level during osteoclast differentiation or formation tend to inhibit osteoclastogenesis, and vice versa. For instance, miR-31 was elevated during RANKL-induced osteoclastogenesis and inhibition of miR-31 impaired the osteoclast formation under RANKL induction [22]. Also, miR-27a attenuates adipogenesis and promotes osteogenesis in steroid-induced rat bone marrow mesenchymal stem cells (BMSCs) by targeting peroxisome proliferator-activated receptor gamma (PPARγ) and gremlin 1 (GREM1) [23]. Despite increasing research on involvement of miRNAs in regulating bone remodeling, the precise role of miR-125a-5p in osteoclastogenesis requires further investigation. Our present study demonstrated that miR-125q-5p can be significantly increased during osteoclast differentiation under the stimulation of M-CSF and RANKL. In addition, the relative mRNA expression levels of TRAP and cathepsin K were increased in RAW 264.7 osteoclast precursor cells that were stimulated by M-CSF and RANKL, which was consistent with the increased TRAP staining activity in cells.

MiR-125a-5p is widely expressed in different tissues and cells. It targets different genes to regulate the occurrence of cancer, immunity, differentiation of skeletal muscle cells, protection of heart from ischemic injury, and inhibition of angiogenesis [24]. In this study, our results demonstrated that miR-125a-5p was significantly increased in RAW 264.7 cells that were treated with M-CSF and RANKL. In addition, up-regulation of miR-125a-5p significantly increased the proliferation, migration and invasion of RAW 264.7 cells in vitro, whereas down-regulation of miR-125a-5p remarkably inhibited the growth and mobility of cells. Meanwhile, the expression levels of TRAP, MMP-2, MMP-9, and cathepsin K were increased in cells that were transfected with miR-125a-5p and were reduced by anti-miR-125a-5p treatment.

MiRNAs have been reported to mediate negative regulation in gene expression after binding to 3′-UTR of a target mRNA by regulating transcript localization, polyadenylation, and translation, which supported our findings that miR-125a-5p exerted an inhibitory effect on the expression of TNFRSF1B by binding to 3′-UTR of TNFRSF1B [25]. Finally, the rescue experiments demonstrated that the inhibitory effect of miR-125a-5p on the proliferation, migration and invasion of RAW 264.7 cells was rescued by down-regulation of TNFRSF1B and the promoting impact of miR-125a-5p on the growth and mobility of cells was neutralized by over-expression of TNFRSF1B. Taken together, we can conclude that overexpression of miR-125a-5p inhibited osteoclast differentiation, which was likely to be mediated by repression of TNFRSF1B expression.

## Conclusions

In summary, the present study proposes the novel role of miR-125a-5p in osteoclast formation and function by regulating TNFRSF1B expression.

## Abbreviations

BMSCs: Bone marrow mesenchymal stem cells
GIT1: G protein-coupled receptor kinase interacting protein 1
GREM1: Gremlin 1
M-CSF: Colony stimulating factor 1
miRNAs: microRNAs
NFATc1: Nuclear factor of activated T cells 1
NF-κB: Nuclear factor-κB
PPARγ: Peroxisome proliferator-activated receptor gamma
RANKL: Receptor activator of nuclear factor kappa-B ligand, NFATc1: nuclear factor of activated T-cells cytoplasmic
TNFRSF1B: TNF receptor superfamily member 1B
TRAP: Tartrate-resistant acid phosphatase
TRAP: Triiodothyronine receptor auxiliary protein, MMP-2: matrix metallopeptidase 2

## References

[1] Kim K, Kim JH, Kim I, Lee J, Seong S, Park YW, Kim N (2015). MicroRNA-26a regulates RANKL-induced osteoclast formation. Molecules and cells.

[2] Zhao H, Zhang J, Shao H, Liu J, Jin M, Chen J, Huang Y. miRNA-340 inhibits osteoclast differentiation via repression of MITF. Biosci Rep. 2017;27. 10.1042/BSR20170302 PMC5518531 28607030

[3] Hujie G, Zhou SH, Zhang H, Qu J, Xiong XW, Hujie O, Liao CG, Yang SE (2018). MicroRNA-10b regulates epithelial-mesenchymal transition by modulating KLF4/KLF11/Smads in hepatocellular carcinoma. Cancer Cell Int.

[4] Feng ZY, Xu XH, Cen DZ, Luo CY, Wu SB (2017). miR-590-3p promotes colon cancer cell proliferation via Wnt/beta-catenin signaling pathway by inhibiting WIF1 and DKK1. Eur Rev Med Pharmacol Sci.

[5] Zhang X, He X, Liu Y, Zhang H, Chen H, Guo S, Liang Y (2017). MiR-101-3p inhibits the growth and metastasis of non-small cell lung cancer through blocking PI3K/AKT signal pathway by targeting MALAT-1. Biomedicine & pharmacotherapy = Biomedecine & pharmacotherapie.

[6] Saito A, Horie M, Ejiri K, Aoki A, Katagiri S, Maekawa S, Suzuki S, Kong S, Yamauchi T, Yamaguchi Y, Izumi Y, Ohshima M (2017). MicroRNA profiling in gingival crevicular fluid of periodontitis-a pilot study. FEBS open bio.

[7] Perri R, Nares S, Zhang S, Barros SP, Offenbacher S (2012). MicroRNA modulation in obesity and periodontitis. J Dent Res.

[8] Zhao Y, Ma K, Yang S, Zhang X, Wang F, Zhang X, Liu H, Fan Q (2018). MicroRNA-125a-5p enhances the sensitivity of esophageal squamous cell carcinoma cells to cisplatin by suppressing the activation of the STAT3 signaling pathway. Int J Oncol.

[9] Cao Y, Tan S, Tu Y, Zhang G, Liu Y, Li D, Xu S, Le Z, Xiong J, Zou W, Gong P, Li Z, Jie Z (2018). MicroRNA-125a-5p inhibits invasion and metastasis of gastric cancer cells by targeting BRMS1 expression. Oncol Lett.

[10] Tang L, Shen H, Li X, Li Z, Liu Z, Xu J, Ma S, Zhao X, Bai X, Li M, Wang Q, Ji J (2016). MiR-125a-5p decreases after long non-coding RNA HOTAIR knockdown to promote cancer cell apoptosis by releasing caspase 2. Cell Death Dis.

[11] Qin X, Wan Y, Wang S, Xue M (2016). MicroRNA-125a-5p modulates human cervical carcinoma proliferation and migration by targeting ABL2. Drug design, development and therapy.

[12] Liang L, Gao C, Li Y, Sun M, Xu J, Li H, Jia L, Zhao Y (2017). miR-125a-3p/FUT5-FUT6 axis mediates colorectal cancer cell proliferation, migration, invasion and pathological angiogenesis via PI3K-Akt pathway. Cell Death Dis.

[13] Tu XM, Gu YL, Ren GQ (2016). miR-125a-3p targetedly regulates GIT1 expression to inhibit osteoblastic proliferation and differentiation. Experimental and therapeutic medicine.

[14] Zhao W, Geng D, Li S, Chen Z, Sun M (2018). LncRNA HOTAIR influences cell growth, migration, invasion, and apoptosis via the miR-20a-5p/HMGA2 axis in breast cancer. Cancer medicine.

[15] Wang P, Liu G, Xu W, Liu H, Bu Q, Sun D (2017). Long noncoding RNA H19 inhibits cell viability, migration, and invasion via downregulation of IRS-1 in thyroid Cancer cells. Technology in cancer research & treatment.

[16] Zhu S, Huang Y, Su X (2016). Mir-451 correlates with prognosis of renal cell carcinoma patients and inhibits cellular proliferation of renal cell carcinoma. Med Sci Monit.

[17] Zhang S, Ge W, Zou G, Yu L, Zhu Y, Li Q, Zhang Y, Wang Z, Xu T (2018). MiR-382 targets GOLM1 to inhibit metastasis of hepatocellular carcinoma and its down-regulation predicts a poor survival. Am J Cancer Res.

[18] Zhao Q, Shao J, Chen W, Li YP (2007). Osteoclast differentiation and gene regulation. Frontiers in bioscience : a journal and virtual library.

[19] Zur Y, Rosenfeld L, Keshelman CA, Dalal N, Guterman-Ram G, Orenbuch A, Einav Y, Levaot N, Papo N (2018). A dual-specific macrophage colony-stimulating factor antagonist of c-FMS and alphavbeta3 integrin for osteoporosis therapy. PLoS Biol.

[20] Lee K, Chung YH, Ahn H, Kim H, Rho J, Jeong D (2016). Selective regulation of MAPK signaling mediates RANKL-dependent osteoclast differentiation. Int J Biol Sci.

[21] Tang P, Xiong Q, Ge W, Zhang L (2014). The role of microRNAs in osteoclasts and osteoporosis. RNA Biol.

[22] Mizoguchi F, Murakami Y, Saito T, Miyasaka N, Kohsaka H (2013). miR-31 controls osteoclast formation and bone resorption by targeting RhoA. Arthritis research & therapy.

[23] Gu C, Xu Y, Zhang S, Guan H, Song S, Wang X, Wang Y, Li Y, Zhao G (2016). miR-27a attenuates adipogenesis and promotes osteogenesis in steroid-induced rat BMSCs by targeting PPARgamma and GREM1. Sci Rep.

[24] Cai Z, Li J, Zhuang Q, Zhang X, Yuan A, Shen L, Kang K, Qu B, Tang Y, Pu J, Gou D, Shen J (2018). MiR-125a-5p ameliorates monocrotaline-induced pulmonary arterial hypertension by targeting the TGF-beta1 and IL-6/STAT3 signaling pathways. Exp Mol Med.

[25] Wang ZM, Wan XH, Sang GY, Zhao JD, Zhu QY, Wang DM (2017). miR-15a-5p suppresses endometrial cancer cell growth via Wnt/beta-catenin signaling pathway by inhibiting WNT3A. Eur Rev Med Pharmacol Sci.

